# Supplementary material for: Towards implementing hierarchical porous zeolitic imidazolate frameworks in dye-sensitized solar cells
Source: R Soc Open Sci. 2019 Jul 10;6(7):190723. doi: 10.1098/rsos.190723 (PMC6689607; doi:10.1098/rsos.190723)
Supplement: Figures S1 - S5 [file rsos190723supp1.docx]

**Electronic Supporting Information**

### Towards Implementing Hierarchical Porous Zeolitic Imidazolate Frameworks in Dye Sensitized Solar Cells

### Hani Nasser Abdelhamid^1,2*^, Ahmed M. El-Zohry^3^, Jiayan Cong^4*^, Thomas Thersleff^1^, Martin Karlsson^4^, Lars Kloo^4^, Xiaodong Zou^1*^

^1^Department of Materials and Environmental Chemistry, Stockholm University, Svante Arrhenius väg 16C, Stockholm, SE-106 91, Sweden

^2^Advanced Multifunctional Materials Laboratory, Department of Chemistry, Assiut University, Assiut, 71515, Egypt

^3^Department of Chemistry, Ångström Laboratories, Uppsala University, Box 523, SE-75120 Uppsala, Sweden

^4^Applied Physical Chemistry, Department of Chemistry, KTH Royal Institute of Technology, Teknikringen 30, SE-10044 Stockholm, Sweden

Correspondence may be addressed to H. N. Abdelhamid. Email: [hany.abdelhameed@science.au.edu.eg](mailto:hany.abdelhameed@science.au.edu.eg); J. Cong. Email: jiayan@kth.se; X. Zou.; Email: [xzou@mmk.su.se](mailto:xzou@mmk.su.se)

**Experimental**

**Preparation of dye@ZIF-8-coated TiO_2_ electrodes**

The nanocrystalline TiO_2_ film deposited on a fluorine-doped tin oxide (FTO) coated glass substrate (2.2 mm thickness, sheet resistance of 15 Ω/cm^2^, TEC 15, Pilkington) was prepared as described in literature ^[1]^.

Three different methods were used to prepare the dye@ZIF-8-coated TiO_2_ electrodes. First method, denoted as *in situ*, is based on the synthesize of dye@ZIF-8 in the presence of FTO glass substrates (Pilkington, TEC15) for 1, 2 and 3h following the same procedure as mentioned above. Second method is direct deposition of L1@ZIF-8 slurry (1 mg was dispersed in 1 mL of methanol) to the chip. Third method is layer by layer. Simply, 1 mL of the dye solution was deposited in the chip. Then, 0.8 mL of Zn(NO_3_)_2_·6H_2_O (0.2 g/0.8 mL H_2_O, 0.7 mmol, 0.84 M) was added following by the addition of TEA (100 μL). The dye solution (1 mL) was deposited flowed by a solution of Hmim (8 mL). Finally, the dye solution (1 mL) was deposited. L1Fc@ZIF-8 deposited TiO_2_ film-FTO was prepared using the same procedure using L1Fc instead of L1. All the chips were washed with ethanol and methanol prior to dry in oven (85 °C).

**Instruments**

Powder X-ray diffraction (XRD) patterns were recorded using PANalytical X’Pert Pro diffractometer equipped with a Pixel detector using Cu K_a1_ radiation (wavelength, 1.54 Å). XRD patterns were recorded using current, accelerating voltage, source silt of 40 mA, 40 V, and 10 mm, respectively. Transmission electron microscopy (TEM) images were carried on JEOL JEM 2100 with accelerating voltage 200 kV. The HAADF image was acquired by operating in Scanning TEM (STEM, JEOL JEM 2100F) mode and using a high angle annular dark field detector. This yields a predominately mass-thickness contrast. A spectrum image using Electron Energy-Loss Spectroscopy (EELS) was acquired and used to generate the elemental maps in this figure. Energy dispersive X-Ray spectroscopy (EDX) was simultaneously acquired and the integrated spectrum from the MOF is presented in Figure 3. Images using scanning electron microscopy (SEM) were recorded using JEOL JSM-7000F at an accelerating voltage of 15.0 kV. N_2_ adsorption-desorption isotherms were recorded at 77 K using Micromeritics ASAP 2020 instrument. Pore size distribution was determined using Barrett-Joyner-Halenda (BJH) method and non-local density functional theory (NLDFT) method (using the model of carbon slit pores). Elemental analysis of sulfur relating to the dye (L1, L1Fc, L1Fc_2_) encapsulated ZIF-8 was measured in MEDAC Ltd, United Kingdom.

Open circuit voltage (V_oc_), short-circuit current density (J_sc_), fill factor (FF), and efficiency (η) [2] were recorded by a Keithley 2400 source/meter at 1 sun (1000 W/m^2^, AM 1.5 G) conditionusing a Newport solar simulator (model 91160), that was calibrated using a certified reference solar cell (FraunhoferISE) to an intensity of 1000 W·m^-2^. The wavelength dependence of the incident photon-current efficiency (IPCE) was measured using light from xenon lamp through monochromator (PXJ43B11, Japan) onto the cell. The IPCE value of the solar cell was calculated according to the equation of Ref. [2]. Electrochemical impedance spectroscopy (EIS) was tested with an EIS600 potentiostat (Gamry Instruments, Warminster, PA, USA).

**Steady state emission and time correlated single photon counting (TCSPC) measurements**

Fluorescence excitation-emission spectra and excitation lifetime at *λ*_max_ 404 nm were recorded using HORIBA JobinYvon Fluorolog 3-222 spectrofluorometer, equipped with a 450 W xenon arc lamp and a R928P PMT detector for the UV-VIS range. The details for TCSPC were describe in detail previously ^[3]^, briefly, laser pulse with IRF ≈ 70 ps and wavelength of 404 nm was used to excite the samples and the collected photons were focused in PMT and the histogram was recorded using time-tag unit.

**b**

**a**

**c**

**Figure S1** FT-IR spectra of a) L1@ZIF-8, b) L1Fc@ZIF-8, and c) L1Fc_2_@ZIF-8.


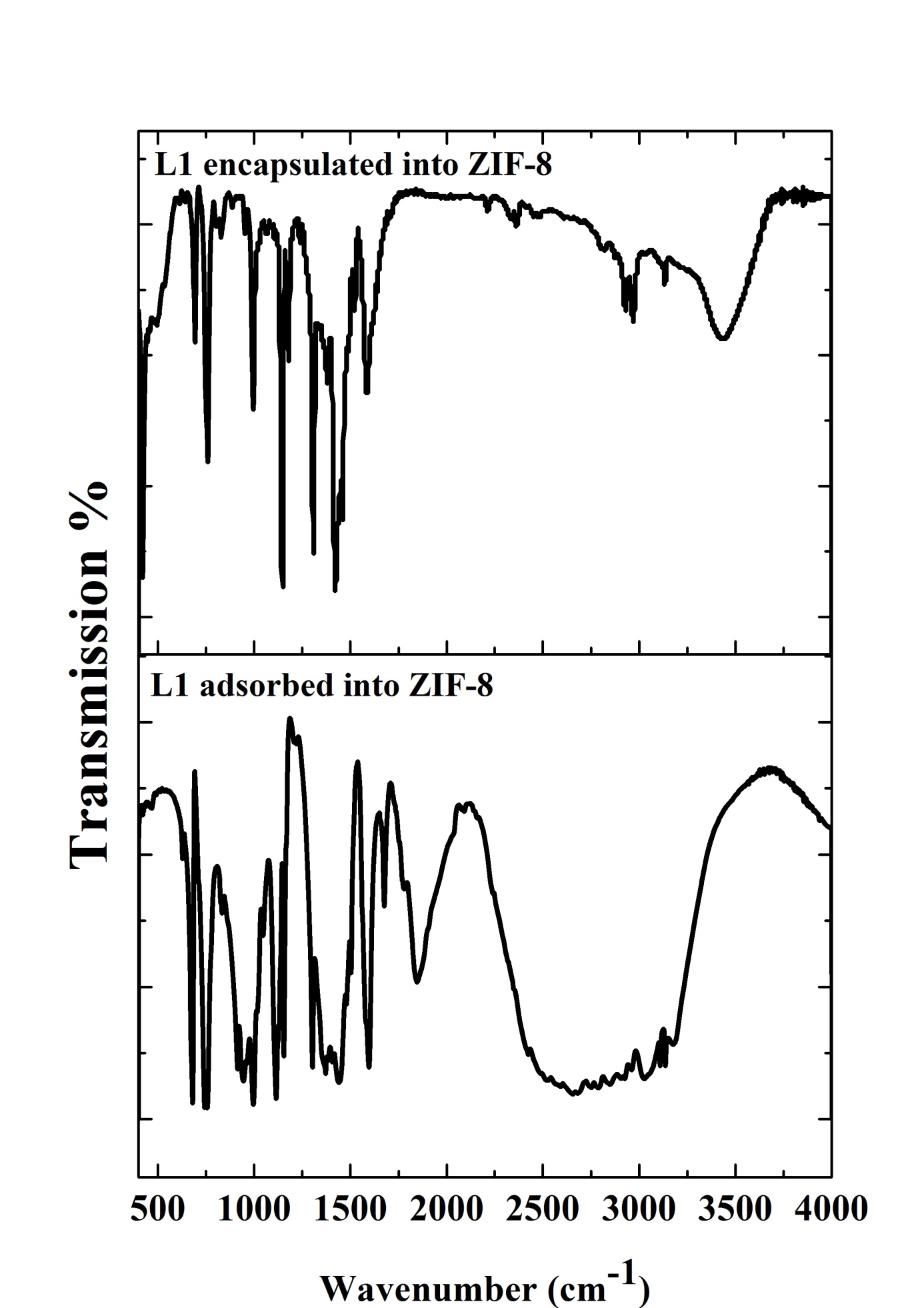


**Figure S2** FT-IR spectra of L1 encapsulated and adsorbed into ZIF-8.





**a**





**b**





**c**

**Figure S3** SEM images of a) L1@ZIF-8, b) L1Fc@ZIF-8, and c) L1Fc_2_@ZIF-8.


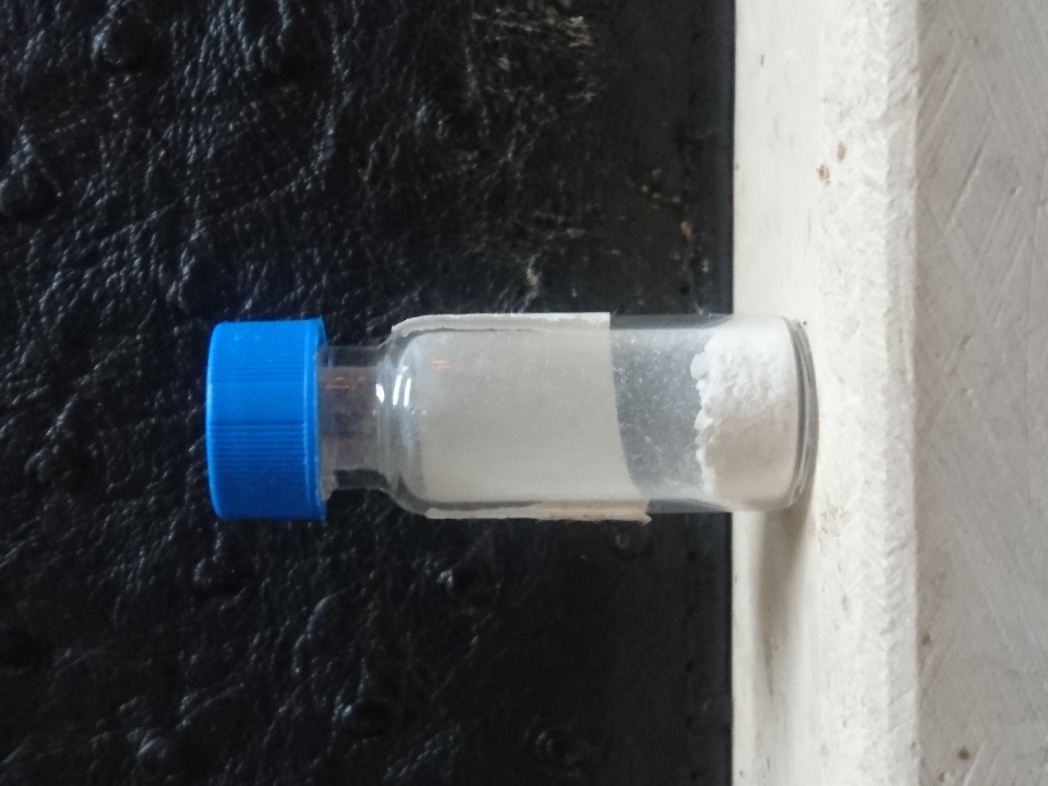

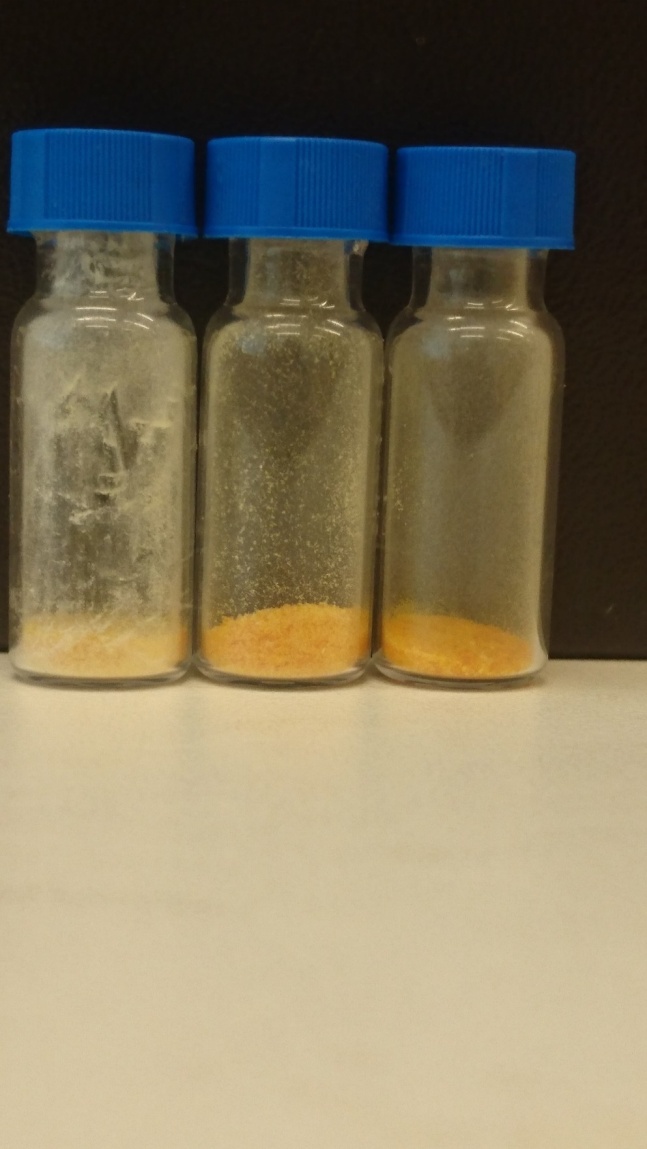


**ZIF-8 L1@ZIF-8 L1Fc@ZIF-8 L1Fc_2_@ZIF-8**

**Figure S4** Camera images of ZIF-8, L1@ZIF-8, L1Fc@ZIF-8, and L1Fc_2_@ZIF-8.

**Figure S5** NLDFT pore size distribution using N_2_@77 on Carbon slit pores.

**Table S1** Surface areas using BET, and Langmuir models, pore volumes, and yields of the prepared.

| **Materials** | **Yield%** | **S_BET_ (m^2^/g)** | **S_Lan_ (m^2^/g)** | **Pore volume (cm^3^/g)** | **Micropore vol. (cm^3^/g)** | **Mesopore Vol. (cm^3^/g)** |
| --- | --- | --- | --- | --- | --- | --- |
| **L1@ZIF-8** | >80 | 450 | 960 | 0.36 | 0.27 | 0.09 |
| **L1Fc@ZIF-8** | >91 | 480 | 1000 | 0.44 | 0.29 | 0.15 |
| **L1Fc_2_@ZIF-8** | >93 | 700 | 1500 | 0.61 | 0.47 | 0.14 |

**Figure S6** Excitation emission spectra of dye solar cell before and after encapsulation into ZIF-8 for a) L1, b) L1Fc and c) L1Fc_2_.

**Figure S7** Camera images of FTO glasses coated by L1@ZIF-8 using a) in situ method (after 3h of the reaction), b) direct deposition, and c) layer by layer.


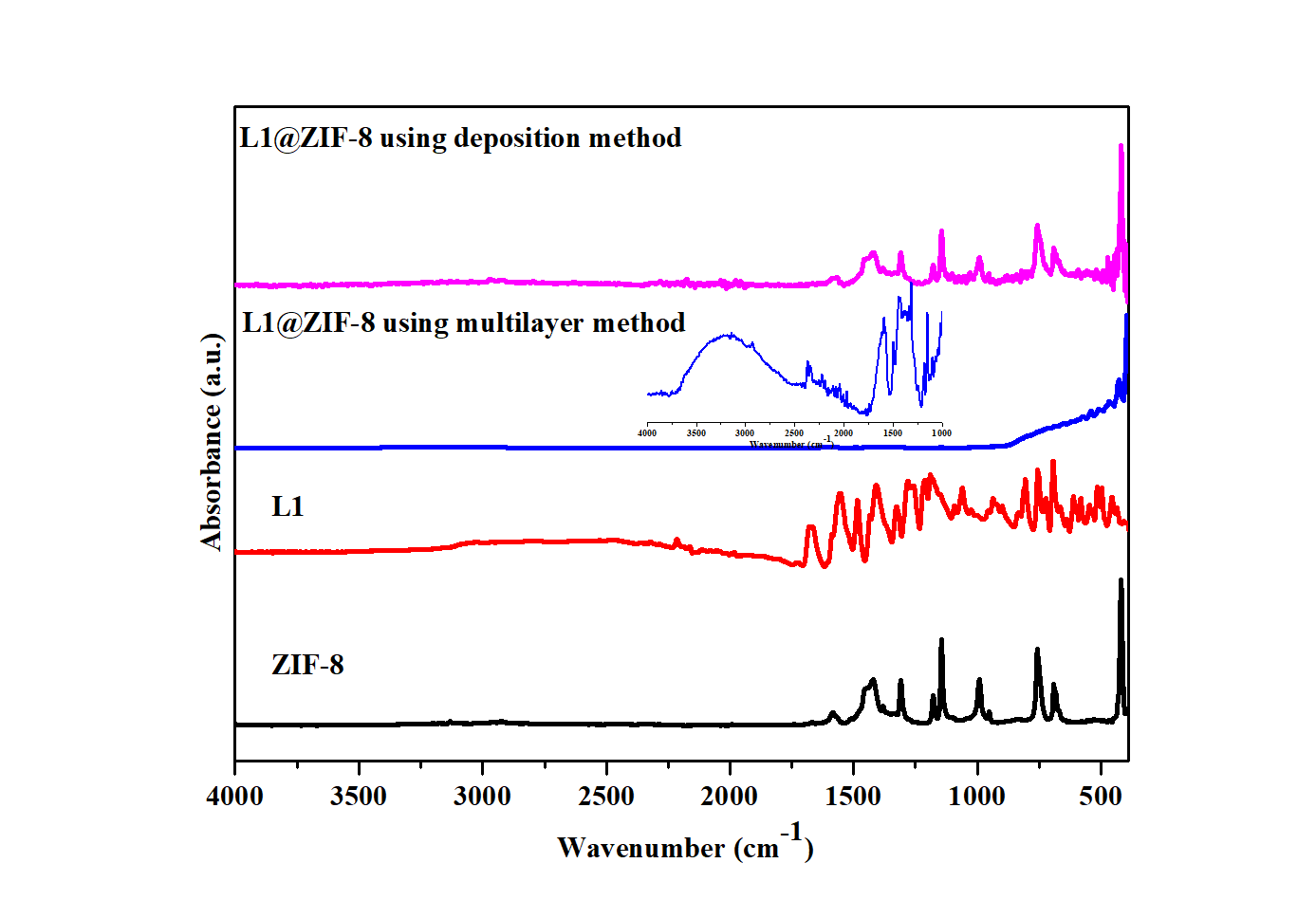


**Figure S8** FT-IR spectra of L1@ZIF-8 on the glasses.









**Figure S9** SEM images of L1@ZIF-8 coated FTO glasses using layer by layer method for different magnifications.


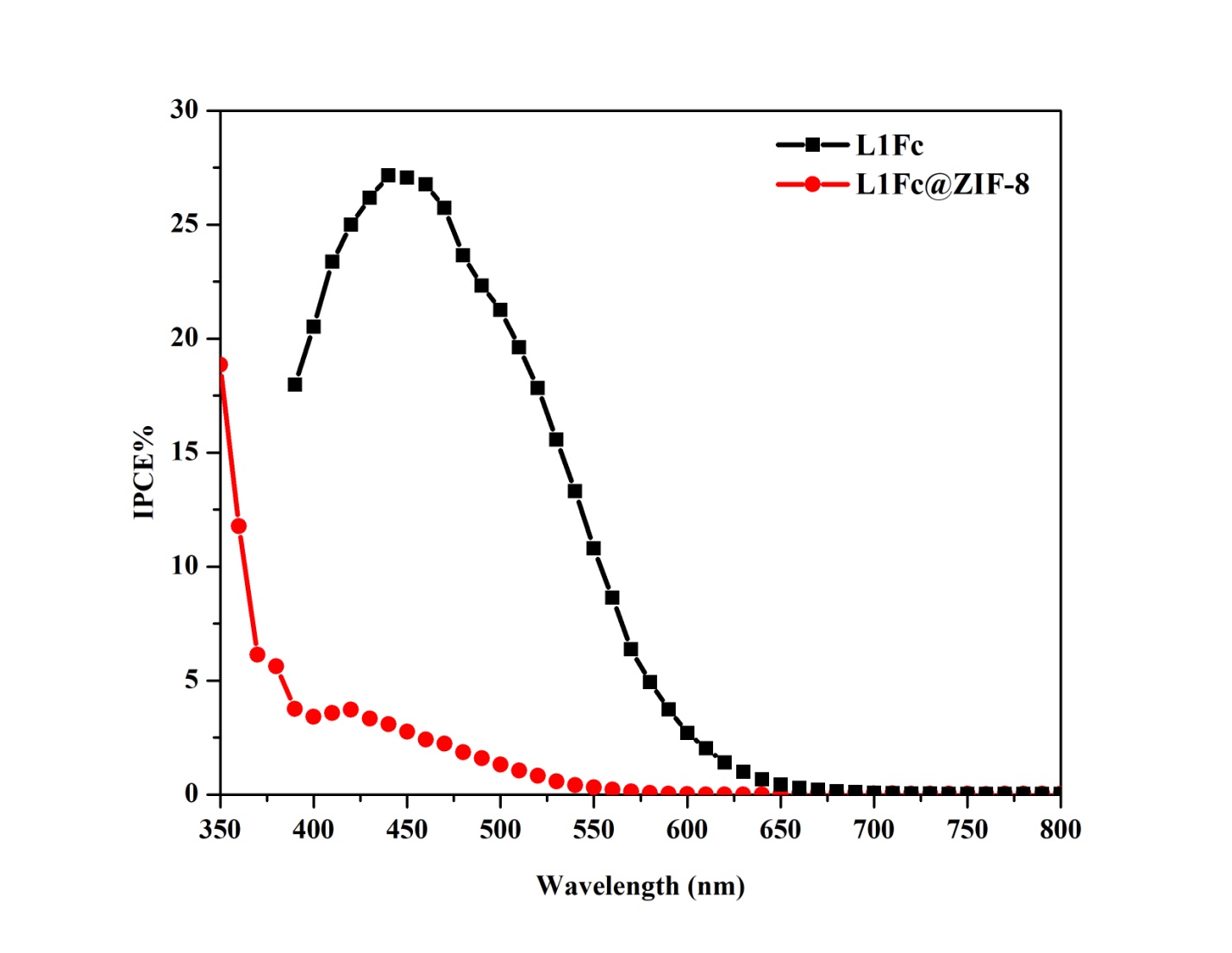


**Figure S10** IPCE spectra for L1Fc, and L1Fc@ZIF-8.


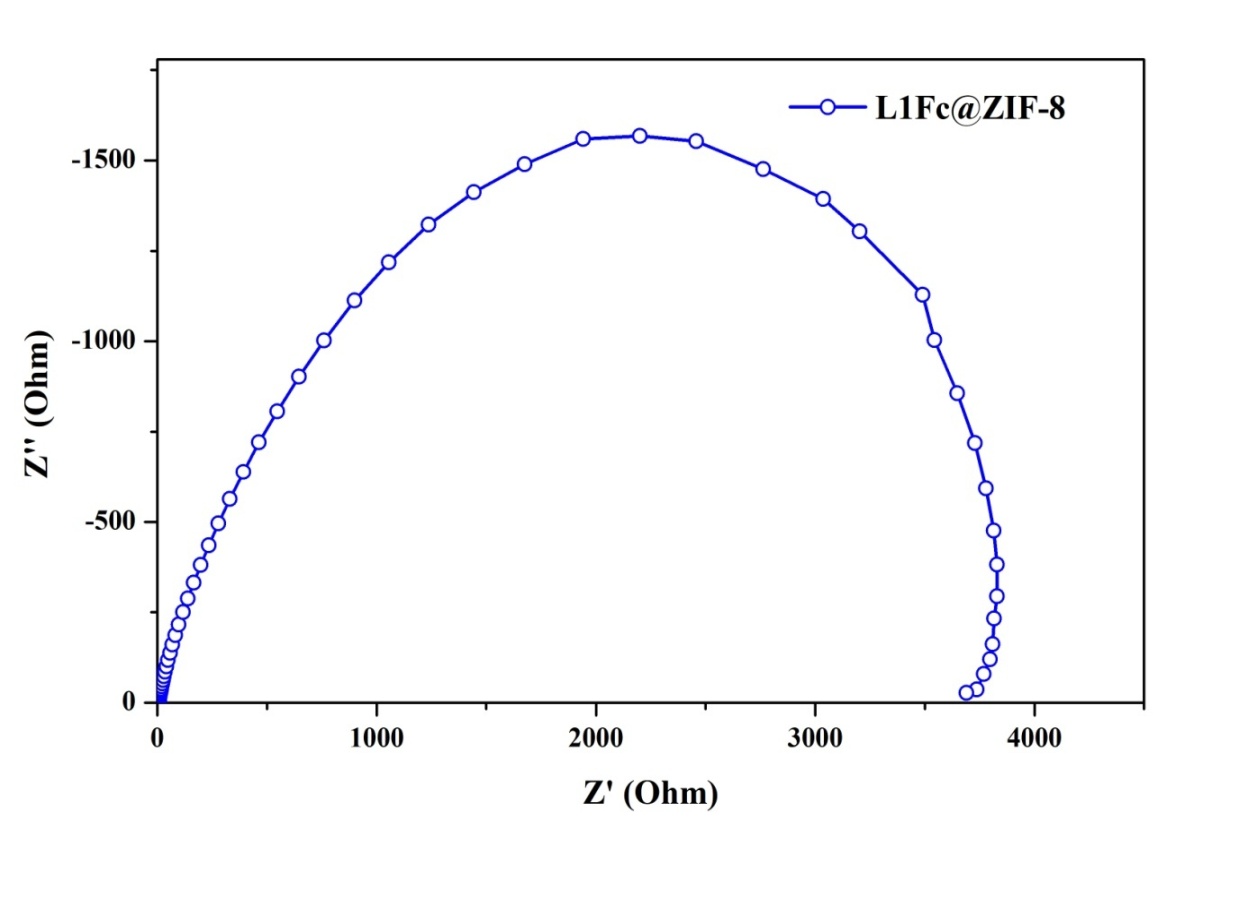


**Figure S11** Nyquist Plot for L1Fc@ZIF-8.

**References**

[1] A. M. El-Zohry, J. Cong, M. Karlsson, L. Kloo, B. Zietz, *Dye. Pigment.* **2016**, *132*, 360.

[2] A. Hagfeldt, G. Boschloo, L. Sun, L. Kloo, H. Pettersson, *Chem. Rev.* **2010**, *110*, 6595.

[3] A. El-Zohry, A. Orthaber, B. Zietz, *J. Phys. Chem. C* **2012**, *116*, 26144.
